# Metachronous Mediastinal Cancers of Unknown Primary: A Case of Sequential Squamous Cell Carcinoma and Adenocarcinoma in Thoracic Lymph Nodes

**DOI:** 10.7759/cureus.86526

**Published:** 2025-06-22

**Authors:** Yasushi Sakamaki, Yuya Kogita, Seigo Minami, Kouji Azuma, Yuuki Kou, Ryosuke Matsuda, Takayoshi Goto

**Affiliations:** 1 Department of Thoracic Surgery, Osaka Keisatsu Hospital, Osaka, JPN; 2 Department of Respiratory Medicine, National Hospital Organization Osaka Medical Center, Osaka, JPN; 3 Department of Diagnostic Pathology, Hannan Municipal Hospital, Hannan, JPN

**Keywords:** mediastinal lymph node metastasis, metachronous cancers, metastatic cancer of unknown primary, pulmonary adenocarcinoma, thoracic lymph nodes

## Abstract

Cancer of unknown primary (CUP) rarely originates in the mediastinum and is even less common as a second malignancy following a prior CUP resection.

A 61-year-old man with no prior malignancy was diagnosed with an anterior mediastinal tumor, resected and identified as metastatic squamous cell carcinoma of unknown primary originating in a lymph node. Eighteen months later, during pneumonia treatment, a computed tomography (CT) scan revealed enlarged lower paratracheal lymph nodes. Initial biopsy showed no malignancy, and the lymph nodes remained stable for 20 months. Over the next year, these nodes progressively enlarged, extending to the subcarinal and left hilar lymph nodes. A transbronchial biopsy confirmed the subcarinal lesion as a poorly differentiated adenocarcinoma, distinct from the initial cancer. A comprehensive systemic workup failed to identify a primary site, leading to a second CUP diagnosis. Due to chronic respiratory failure from underlying lung disease, the patient was ineligible for cancer treatment. One year after the second CUP diagnosis, a CT scan detected a mass in the right lower lobe, which grew over the following year, as confirmed by a follow-up CT scan, suggesting a hidden lung cancer that emerged later. The patient passed away 75 months after the first cancer surgery and 24 months after the second CUP diagnosis, two weeks after the final scan, without an autopsy.

This case highlights the rare occurrence of sequential, histologically distinct CUPs - likely represents the first reported instance - and the diagnostic challenges in identifying primary cancer sites, compounded by the patient’s comorbidities precluding treatment.

## Introduction

Cancer of unknown primary site (CUP) is a prevalent malignancy, ranking among the top 10 most diagnosed cancers in developed societies, and it frequently manifests as metastatic lymph node cancer [1]. While nodal metastatic CUP infrequently originates in the mediastinum [2, 3], it is even rarer for such mediastinal tumors to emerge as a second cancer, extending to the hilar lymph nodes, following the resection of a prior CUP. In this report, we describe a case of CUP presenting as multiple lesions in the mediastinal and hilar lymph nodes, occurring several years after the resection of a previous, histologically distinct CUP in the mediastinum.

## Case presentation

**Figure 1:**
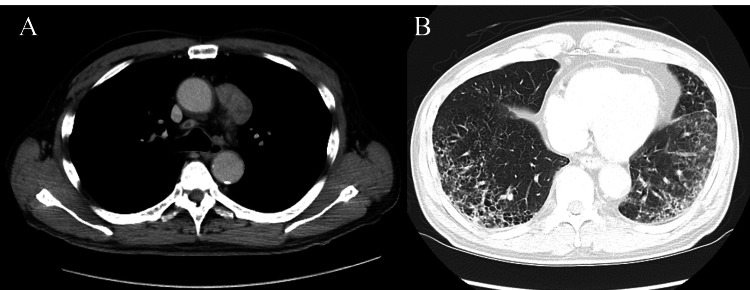
Computed tomography showing the first cancer. An anterior mediastinal tumor (A) alongside coexisting combined pulmonary fibrosis and emphysema (B) was revealed. The tumor, measuring 4.0 × 2.0 cm², appeared noninvasive.

A 61-year-old man with no prior history of malignancy was incidentally found to have an anterior mediastinal tumor, which was suspected to be a noninvasive thymic epithelial tumor based on computed tomography (CT) findings (Figure 1).

**Figure 2:**
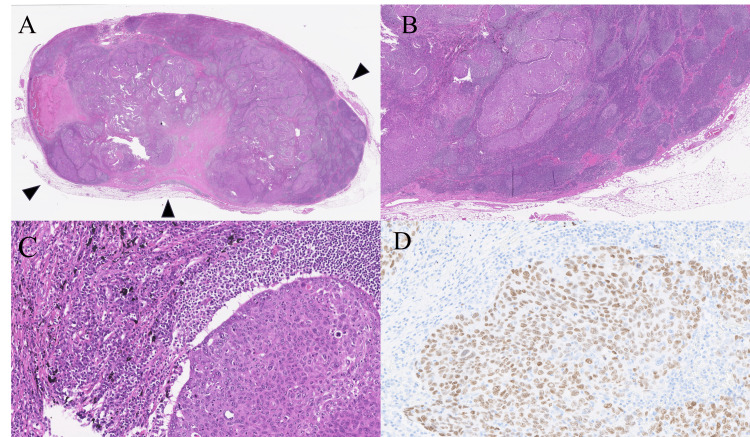
Histological findings and immunoreactivity of the first cancer. Hematoxylin–eosin staining displayed proliferation of atypical squamous cells with (A) capsules (arrowheads), (B) lymphoid follicles containing germ cell centers, and (C) charcoal powder deposits, (D) immunoreactivity to p40 was also observed.

This CT scan was performed to screen for cancer due to an elevated serum carcinoembryonic antigen (CEA) level of 13.2 ng/mL (normal range <5.0 ng/mL), identified during a medical checkup, and to monitor known combined pulmonary fibrosis and emphysema (CPFE). The patient underwent thoracoscopic thymectomy to remove the tumor, and pathological examination identified the lesion as squamous cell carcinoma, not of thymic origin, as determined by histological and immunohistochemical staining (IHC) results (Figure 2).

**Figure 3:**
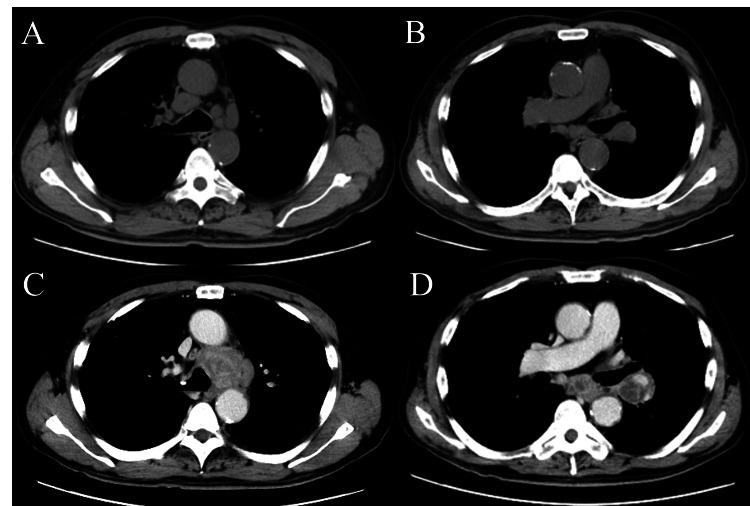
Computed tomography showing enlarged mediastinal lymph nodes at different time points. (A) and (B) were taken 18 months after surgery for the first cancer, while (C) and (D) were captured 51 months post-surgery, coinciding with the diagnosis of the second cancer. Images A and C depict the lower paratracheal region, and B and D show the subcarinal to hilar region.

The tumor contained no thymic tissue but exhibited lymph node-like features-such as capsules, lymphoid follicles with germinal centers, and charcoal powder deposits-and showed a negative reaction to cluster of differentiation 5 (CD5) and type III receptor tyrosine kinases (c-kit). Positive staining for p40 and negative staining for thyroid transcription factor 1 (TTF-1) confirmed it as squamous cell carcinoma. Despite a thorough diagnostic workup planned in anticipation of a malignancy, no other lesions or a primary site could be identified, leading to a diagnosis of nodal metastatic CUP. A month after surgery, the serum CEA level decreased to 9.0 ng/mL, reflecting a fluctuation consistent with the tumor burden, despite the tumor's non-adenocarcinoma histology. Adjuvant chemotherapy was not pursued due to the uncertainty surrounding the primary cancer type and the risk of worsening the patient’s underlying CPFE. Eighteen months after his surgery, the patient developed pneumonia, and a CT scan revealed enlarged mediastinal lymph nodes, predominantly in the lower paratracheal region (Figures 3A-3B).

**Figure 4:**
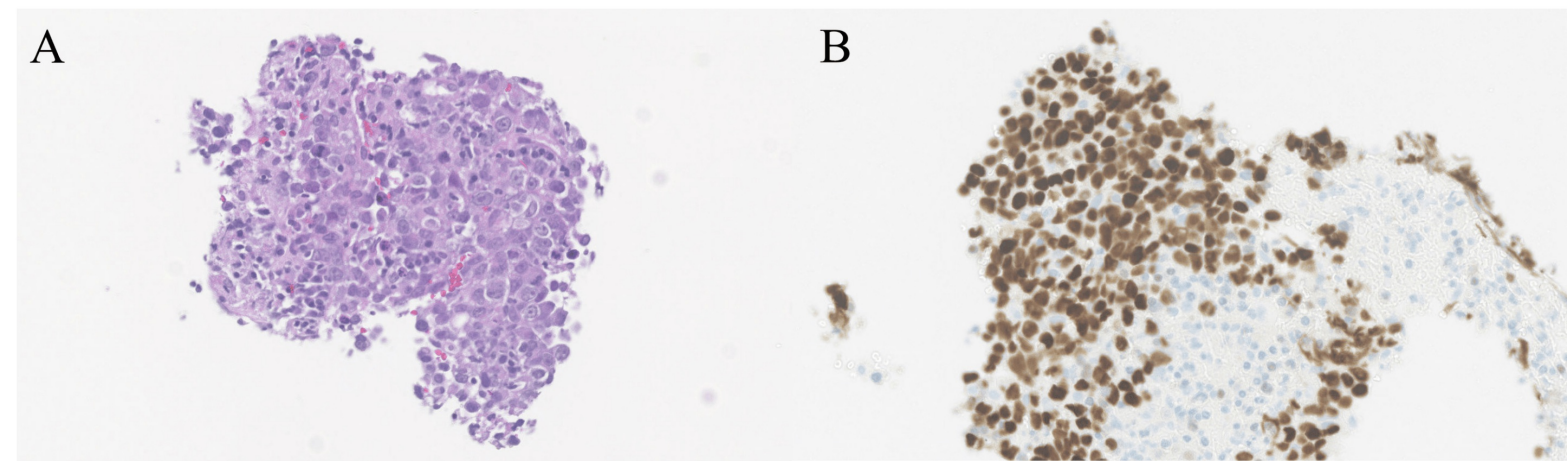
Histological findings and immunoreactivity of the second cancer. (A) Hematoxylin–eosin staining revealed poorly differentiated adenocarcinoma. Immunoreactivity was noted for (B) thyroid transcription factor 1.

The CEA level rose to 13.3 ng/mL, suggesting tumor recurrence despite the non-adenocarcinoma histology, given the case-specific fluctuation in serum CEA observed previously. An endobronchial ultrasound-guided transbronchial needle aspiration (EBUS-TBNA) performed at the time showed no evidence of malignancy, and since the lymphadenopathy remained stable in size over the next 20 months, it was classified as reactive, with no other lesions revealed during the above period. However, over the following 13 months, these lymph nodes gradually enlarged into masses and the lymphadenopathy progressed, eventually extending to the subcarinal and the left hilar lymph nodes (Figures 3C-3D). At that time, serum CEA level was measured at 7.2 ng/mL, showing a decrease over the past 33 months. A subsequent EBUS-TBNA of the subcarinal lymph node confirmed the presence of a poorly differentiated adenocarcinoma, with IHC findings consistent with a lung cancer profile (Figure 4).

Specifically, positive staining for TTF-1 and cell adhesion molecule 5.2 (CAM 5.2), along with negative staining for p40, strongly supported a diagnosis of lung adenocarcinoma, distinguishing it from squamous cell carcinoma. As with the patient’s prior cancer, a comprehensive systemic evaluation failed to identify the primary site of this lymph node cancer. Consequently, the patient was diagnosed with a second CUP, distinct in histological type from the initial cancer. Serum tumor markers for lung cancer, including CEA, were infrequently measured and consistently elevated, yet their fluctuations did not correspond with specific tumor progression typically associated with each marker (Table 1).

**Table 1:** Laboratory test for serum tumor markers at different time points. CEA: carcinoembryonic antigen; CYFRA: cytokeratin 19 fragment; Pro-GRP: pro-gastrin-releasing peptide. *Paratracheal lymph nodes were enlarged but confirmed negative. **Second cancer (adenocarcinoma) was detected. ***Lung tumor appeared.

			Time After Surgery (Months)		
Tumor marker (Normal range)	Before surgery	1	18*	51**	63***
CEA (< 5.0 ng/mL)	13.2	9	13.3	7.2	4.6
CYFRA (0.3-2.1 ng/mL)	Not tested	Not tested	7.4	11.7	8.3
Pro-GRP (< 80.9 pg/mL)	Not tested	Not tested	47.2	58.1	130

**Figure 5:**
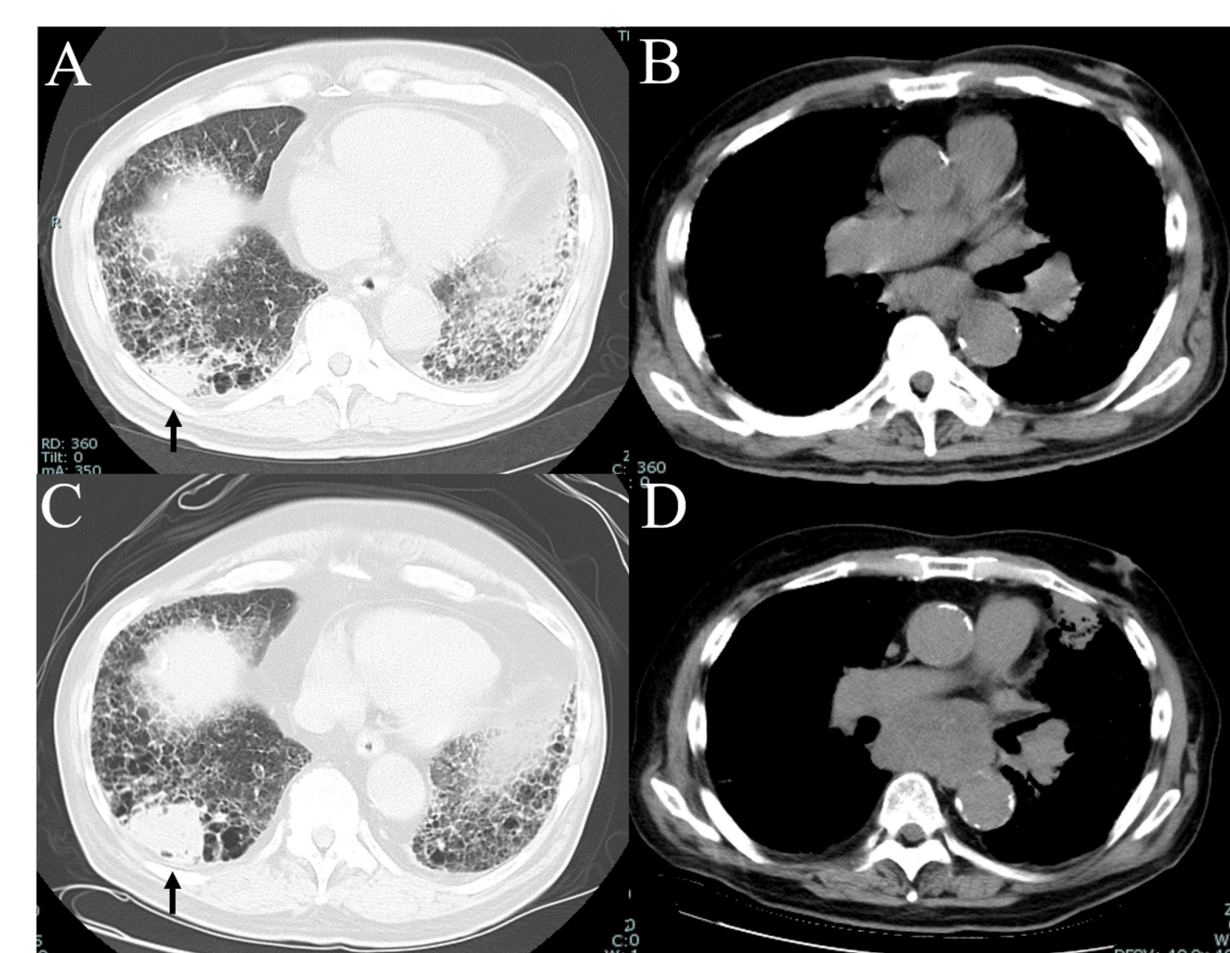
Computed tomography showing a lung mass and enlarged mediastinal lymph nodes at different time points. (A) and (B) were taken 12 months after the diagnosis of the second cancer, with a newly developed lung mass in the right lower lobe, while (C) and (D) were captured 24 months post-diagnosis. Images A and C depict the lung mass (arrows), and B and D show the subcarinal region.

Due to chronic respiratory failure from CPFE, the patient was unable to receive cancer treatment for the second CUP. Instead, he was managed with pirfenidone and home oxygen therapy. He opted for supportive care for his cancer and declined resuscitation attempts for any life-threatening conditions. A year after the above diagnosis, however, a CT scan detected a mass in the right lower lobe (Figure 5).

A follow-up CT scan a year later showed the mass had grown, indicating a likely hidden lung cancer that emerged later. No biopsy was available due to the patient’s severe respiratory failure, which precluded clarification whether this lung lesion represented a later surfacing occult primary or a distinct lesion not associated with his CUPs. He passed away two weeks after this final scan and 24 months after the second cancer diagnosis (75 months after surgery for the first cancer). No autopsy was performed.

## Discussion

This case is exceptionally rare due to its distinctive features. First, it presents an unusual clinical history characterized by two distinct episodes of CUP, termed 'metachronous CUPs,' occurring sequentially over time-here better described as 'CUP after CUP.' While CUP accounts for 3%-5% of all malignancies [1], the development of two independent CUPs is presumed rare, though epidemiological data are lacking. A PubMed search (using terms such as “metachronous CUP”, “thoracic lymph node CUP”, and “multiple CUPs”) conducted on May 17, 2025, identified no prior reports of metachronous thoracic lymph node CUP, and existing literature offers limited insight into such occurrences [4-6]. Second, the natural history of untreated CUP in this patient is notable. Typically, patients with poor-prognosis CUP, such as those with unresectable mediastinal lymph node involvement, have a median survival of less than 12 months, even with chemotherapy [7]. In contrast, this patient, despite a concurrent diagnosis of CPFE-which posed a constant risk of fatal respiratory failure-survived for two years without treatment following the second CUP diagnosis, markedly exceeding expected outcomes.

In this case, IHC played a pivotal role in distinguishing the two instances of CUP, supporting the diagnosis of metachronous CUPs rather than a revised classification of a single tumor. While it is not uncommon in clinical practice for an initial diagnosis of poorly differentiated adenocarcinoma to be reclassified as squamous cell carcinoma based on further testing, here, IHC profiles confirmed distinct tumor identities. The first CUP showed features consistent with a squamous cell carcinoma, including p40 positivity, alongside histological evidence of lymph node involvement, whereas the second exhibited markers (e.g., TTF-1, CAM 5.2) typical of lung adenocarcinoma, suggestive of N2 disease with an occult primary. This distinction aligns with the patient’s history of CPFE, a condition associated with an elevated risk of lung cancer, particularly adenocarcinoma and squamous cell carcinoma [8]. Whether CPFE likely contributed to the oncogenic milieu is unknown with the absence of a detectable primary site despite extensive evaluation, though a lung mass found a year after the second CUP diagnosis represented a likely hidden primary that emerged later. A study in Europe reported a relatively high incidence of lung cancer as a subsequent primary cancer following CUP, with standardized incidence ratios ranging from 2.07 to 2.76, varying by follow-up period [5]. Another study revealed that lung cancer, as a second primary cancer, occurs more frequently following CUP than after other primary cancers, with age-standardized rate ratios of 7.69 for CUP compared to 2.51 for other cancers, based on nationwide analyses [6]. Additionally, over 10 cases in Japan have been reported where CUP in the thoracic lymph nodes was later identified as lung cancer following treatment [9]. Regardless, it raises the possibility that an autopsy might have confirmed this lesion to be a primary site of either CUP, likely the second one, given the incidence of occult primary lesions that become evident while alive is reportedly 20% to 40% of all CUP cases [1, 10]. Conversely, this lung mass might have also been a pulmonary metastasis from either CUP or a distinct lesion unrelated to the preceding CUPs. These findings underscore the diagnostic complexity of metachronous CUPs in this unique case.

This case provided a unique opportunity to observe the untreated natural history of a thoracic lymph node CUP from diagnosis to death, a scenario infrequently documented due to the typical pursuit of treatment in such cases. The duration of the second CUP’s presence is uncertain due to varying definitions of onset, but retrospective analysis of the enlargement process suggests that carcinogenesis or malignant transformation in the paratracheal to subcarinal lymph nodes may have started at least one year before biopsy detected adenocarcinoma in these nodes. Based on the following observations, we propose that the patient’s underlying CPFE, rather than the cancer itself, predominantly shaped his prognosis. First, although the second CUP progressively enlarged over time without symptomatic distant metastases - spanning over the left lower paratracheal, subcarinal, and left hilar regions by the patient’s death - it did not strict the central airways or induce malignant pleural effusion, both of which would have precipitated acute respiratory distress. Second, no evidence of recurrence was observed following surgical removal of the initial CUP, suggesting that it remained clinically dormant. We hypothesize that, in the absence of CPFE, the second CUP’s relatively indolent course might have delayed the onset of respiratory failure, potentially enabling the patient to tolerate cancer-directed therapy and achieve a longer survival. However, the severe pulmonary compromise from CPFE likely limited both treatment options and life expectancy.

In this case, the feasibility of completely resecting the second CUP remains uncertain, as the patient’s severe CPFE likely precluded definitive surgery, even with induction chemotherapy to reduce tumor burden. Radical surgery for CUP confined to the mediastinum, though feasible even in selected cases requiring extensive resection, is rarely curative and yields variable outcomes depending on histology [11-14]. For example, we previously described two patients with mediastinal adenocarcinoma of unknown primary who underwent multimodality therapy, including surgery, and achieved survival exceeding typical expectations [11, 12], whereas two cases of mediastinal large cell neuroendocrine carcinoma treated similarly succumbed to death within 4-5 months due to rapid recurrence [13, 14]. These contrasting outcomes highlight the prognostic heterogeneity of CUP and the limitations of aggressive surgery in poor-prognosis subtypes. In this context, the patient’s second CUP, identified as lung adenocarcinoma-like cancer via IHC, might have warranted consideration of surgery in a less compromised individual, though extensive spanning of the tumor and comorbid CPFE rendered such an approach untenable. Looking ahead, advances in comprehensive genomic profiling, such as next-generation sequencing, offer new avenues for CUP management by inferring primary tumor types and guiding molecularly targeted therapies [15]. As personalized treatments such as immune checkpoint inhibitors gain traction, curative surgery may increasingly be reserved for rare cases where systemic therapies fail, potentially refining treatment strategies and improving outcomes for future CUP patients.

## Conclusions

We documented an exceptionally rare case-potentially the first-of metachronous duplication of CUPs, both originating in the thoracic lymph nodes. If the patient had not been affected by severe underlying lung disease, treatment options for the second CUP could have been pursued, likely resulting in improved survival.
